# The Awareness and Adoption of UK Physical Activity Guidelines by Socio-Demographics: A National Cross-Sectional Survey in Wales

**DOI:** 10.3390/ijerph23010005

**Published:** 2025-12-19

**Authors:** Catherine A. Sharp, Karen Hughes, Paul Pilkington, John Bradley

**Affiliations:** 1 Policy and International Health, a World Health Organization Collaborating Centre on Investment for Health & Well-being, Public Health Wales, Cardiff CF10 4BZ, UK; karen.hughes18@wales.nhs.uk; 2 School of Health Sciences, Bangor University, Wrexham LL13 7YP, UK; 3 Health Improvement Division, Public Health Wales, Cardiff CF10 4BZ, UK; paul.pilkington@wales.nhs.uk (P.P.); john.bradley@wales.nhs.uk (J.B.)

**Keywords:** moderate physical activity, vigorous physical activity, muscle-strengthening activity, policy, surveillance

## Abstract

Designing and communicating physical activity guidelines takes considerable resources; thus, understanding the awareness and adoption of such guidelines by different population groups is important. A national cross-sectional survey (*N* = 972; aged 19+ years living in Wales) was delivered as part of a population panel using a multi-method approach (online, telephone and face-to-face). The survey measured the awareness and adoption of the 2019 UK physical activity guidelines and recommendations and socio-demographics, including age, sex, residential deprivation and disability status. Around a fifth (21.7%) of participants had heard of the physical activity guidelines. Almost a third (30.7%) reported knowing the moderate physical activity recommendation, with 13.3% knowing the vigorous physical activity recommendation and 13.4% knowing the muscle-strengthening recommendation. There were no significant socio-demographic differences in knowing the moderate recommendation (*p* > 0.05); however, females were less likely than males to report knowing the vigorous recommendation (*p* = 0.009), and participants with a disability were less likely than those without a disability to report knowing the muscle-strengthening recommendation (*p* = 0.026). Having heard of the physical activity guidelines increased the likelihood of knowing each of the three recommendations (all *p* < 0.001). Additionally, for both moderate-to-vigorous physical activity and muscle-strengthening recommendations, a positive relationship was found between knowing the recommendation and reporting meeting the recommendation (*p* = 0.008 and *p* = 0.002, respectively). The awareness of both the physical activity guidelines and their recommendations was low. The development of communication strategies to aid knowledge mobilisation should be considered. Socio-demographic differences in awareness should be considered when designing interventions in line with proportionate universalism principles.

## 1. Introduction

In 2021, seven of the top ten causes of death globally were non-communicable diseases [1]. One approach to combat the prevalence and support the management of non-communicable diseases is through increasing physical activity [2,3]. Unfortunately, despite an extensive range of national and international actions to promote physical activity, the global prevalence of physical inactivity is estimated to have increased from 23.4% in 2000 to 31.3% in 2022 [4]. In 2018, the World Health Organization (WHO) published its mandate to reduce physical inactivity in adults by 15% by 2030 [5]. To reach this target, the WHO highlighted the development, implementation and dissemination of physical activity guidelines as a key approach. Physical activity guidelines are important as they provide thresholds for the frequency, duration, intensity, type and quantity of physical activity for the population to complete to experience optimal health benefits [6].

While the earliest physical activity guidelines are proposed to have been published in 1975 by the American College of Sports Medicine, these had a clinical health focus rather than a population health focus [7]. The first WHO guidelines with a global reach were published in 2010 [8] and subsequently updated in 2020 [9]. The goals of the WHO guidelines include reducing inequalities and encouraging everyone to move more to improve overall health outcomes. The WHO advocates for countries to produce their own national guidelines [10]. In 2011, the United Kingdom (UK) was among the first set of nations to publish its own guidelines, which were created collectively by the Chief Medical Officers (CMOs) for Wales, England, Scotland and Northern Ireland [11]. Similarly to the WHO, the UK subsequently updated and published revised guidance in 2019 [6], with a further update expected around 2028. A comparative analysis covering the period up to April 2018 found that 23 of the 28 European Union member states had published physical activity guidance, with 14 nations including information on the duration, intensity and frequency of physical activity guidelines consistent with those of the WHO, as a minimum [12]. While some message framing in the WHO and UK guidelines is different, the minimum requirements of physical activity needed for optimum health are consistent. The current UK guidelines emphasise that ‘*any activity is better than none, and more is better still*’ and recommend that adults aged 19+ years should undertake a weekly minimum of 150 min of moderate-intensity activity, 75 min of vigorous-intensity activity or a combination of both [6]. They also advocate for adults to undertake muscle-strengthening activities at least twice a week. The guidelines target professionals.

Despite the publication of physical activity guidelines, evidence from multiple countries suggests that the public awareness and adoption of such guidelines is low. Research from the USA found that only 9.2% of adults were aware of the existence of national physical activity guidelines, and only 2.9% could accurately identify the guideline as ≥150 min a week [13]. Similar results have been seen in Canadian [14] and British [15] samples, with 12.9% and 17.1% of adults, respectively, being able to accurately identify national guidelines. However, in contrast, a study in Saudi Arabia found that 48.4% of adults reported an awareness of national guidelines, and 38.1% could identify the minimum recommended duration [16]. Poor awareness of physical activity guidelines is a cause for concern, as studies have found that awareness of guidelines is associated with increased activity levels, a likelihood of meeting the recommended minutes of activity and increased energy expenditure [15,16,17]. Further, the influence of physical activity guideline awareness on physical activity can be mediated by health beliefs and health literacy [17], highlighting the importance of communicating guidelines in an accessible way. Recommendations for communicating about physical activity guidelines, informed by participatory workshops [18] and a review [19], have been published to support this action. The target audiences of physical activity guidelines are predominantly policymakers, practitioners, professionals and researchers, with the intention that these professional groups will tailor the communication for the general public groups that they work with [9]. However, guidelines targeting the general population do exist [20].

Understanding differences in the awareness and adoption of physical activity guidelines by socio-demographics (e.g., sex, age, socio-economic status and disability status) is important to prevent further increases in inequalities as some groups gain knowledge that others do not possess. There are well-known inequalities in physical activity, with people living in higher socio-economic environments being more likely to be more physically active [21]. Therefore, it is not surprising that adults with lower education and those who are economically inactive have been found to be less knowledgeable of physical activity guidelines [15]. There is a need to ensure that any communication strategies about physical activity guidelines do not further increase health inequalities and that campaigns are tailored to specific target audiences to be effective.

This study sought to investigate the national awareness of the UK physical activity guidelines and the adoption of its recommendations for adults in a general population sample in Wales, UK, to identify relationships with socio-demographics (i.e., age, sex, deprivation, ethnicity and disability status) and to measure the relationship between knowledge of the guidelines and self-reported adherence to the recommendations.

## 2. Methods

### 2.1. Study Design and Sample

This study uses data collected as part the Time to Talk Public Health (TTPH) Panel, a nationally representative panel of people aged 16+ years who live in Wales (UK) [22]. TTPH was established by Public Health Wales in 2022 to inform public health policy and practice through regular survey engagement with the public. The TTPH panel maintains a sample of around 2500 people, with survey sample targets of 1000 participants. The inclusion criteria are living in Wales (maximum of one person per household), being aged 16 years and over and being cognitively able to participate. A stratified quota sampling approach is used for recruitment, and quota sampling is used for the delivery of each survey. The quotas applied are (i) geography (Welsh health board area), (ii) age, (iii) sex, (iv) deprivation quintile (as determined by the Welsh Index of Deprivation; WIMD [23]) and (v) ethnicity (based on mid-2020 population estimates [24]). The sampling quota design includes an over-sampling of younger people, residents of more deprived areas and ethnic minorities as these groups are often less represented in survey data. The initial panel recruitment used a multi-method, multi-step approach involving telephone, face-to-face, social media advertising and dissemination through contacts [22]. A market research company (MRC) was commissioned to manage the panel and collect the survey data.

All panel members were invited to complete the April 2023 survey via their pre-selected method: online or telephone. Demographic gaps in the quota samples were monitored throughout the 4-week data collection period, and email reminders and chaser calls were issued to panel members accordingly. An additional 100 face-to-face interviews were undertaken with 16–29-year-olds. This approach is used in each survey wave to increase the representation of younger participants, with interviewees invited to become panel members following the completion of the survey. The panel consisted of 2526 members at the launch of the April 2023 survey; 32% of the panel sample participated in the survey. A further 239 new participants also took part in the survey. In total, 1051 participants aged 16+ years completed the survey. As the UK adult physical activity guidelines only apply to people aged 19 years and over, the age criteria for inclusion in this study was set as 19 years and over. The final sample was 972 participants (Supplementary Table S2). Of the included sample, 78.1% took part online, 15.1% took part by telephone and 6.8% took part face-to-face.

### 2.2. Questionnaire

TTPH questionnaires include a range of topics based on organisational need and are developed in collaboration with topic leads in Public Health Wales using existing and validated questions where possible. This study uses data on physical activity collected between 3rd and 30th April 2023, with socio-demographic data collected on the date participants were recruited to the panel (November 2022–April 2023). Supplementary Table S1 provides an outline of the questions, response options and data categorisations for analysis.

Participants were first asked if they had heard of the UK adult physical activity guidelines (response options: yes or no). They were then asked if they were aware of the specific recommendations for moderate physical activity (MPA), vigorous physical activity (VPA) and muscle-strengthening activities (response options: yes—I knew this recommendation; yes—I had a vague idea of this recommendation; no—I did not know this recommendation; or don’t know). Subsequently, participants were asked to report if, over the past week, they had achieved the moderate-to-vigorous physical activity (MVPA) recommendation and the muscle-strengthening activity recommendation (response options: yes; no; or don’t know). Prefer not to say was a response option available for all questions.

The socio-demographic measures included in this study are age, sex, residential deprivation quintiles, ethnicity and disability status. Age was calculated from participants’ date of birth and categorised into four age groups (19–35; 36–50; 51–64; and 65+ years). The residential deprivation quintile was assigned by the MRC using participants’ home postcode and the WIMD 2019. UK census categories were used to collect ethnicity data, which were coded into white [including ethnic minority white] and other than white ethnicity due to low numbers across categories other than white. Participants were defined as having a disability in line with the Equality Act; if they had a condition for 12 months or more that affected their day-to-day activities [25].

### 2.3. Statistical Analysis

Statistical analyses were conducted in SPSS v. 24. The analysis criteria for this study required participants to have valid responses to all outcome (prefer not to say responses were excluded) and socio-demographic variables. Sex was limited to respondents who responded male or female due to low numbers in the ‘other’ category; thus, respondents who reported ‘other’ were excluded. Chi-square tests were used to measure bivariate relationships between outcome measures and participant socio-demographics. Binary logistic regression (enter method) was used to measure independent relationships between outcome measures and participant socio-demographics and perceived knowledge. With low numbers in the other than white ethnicity category and no significant bivariate relationships between ethnicity and outcome variables (Supplementary Table S3), ethnicity was not included in multivariate analyses. Results are presented as adjusted odds ratios (AORs) with 95% confidence intervals (95% CIs) and *p*-values. Significance is defined as *p* < 0.05.

## 3. Results

The analytical sample included 972 participants, of which 69.9% were female. Nearly half the sample (48.7%) was aged 19–50 years, with the remaining 51.3% aged 51+ years, and 97.6% were of a white ethnicity (including ethnic minority white). Across deprivation quintiles, proportions ranged from 16.7% (quintile 1—most deprived) to 22.1% (quintile 4). A demographic breakdown of the sample is provided in Supplementary Table S2.

### 3.1. Awareness of UK Physical Activity Guidelines

Around one fifth of participants (21.7%) reported having heard of the UK physical activity guidelines (Table 1). Bivariate and multivariate analyses found significant associations between deprivation and having heard of the guidelines. The proportion of participants having heard of the guidelines ranged from 28.4% of those living in deprivation quintile 4 (second least deprived) to 14.8% of those living in the most deprived quintile; however, only 16.8% of those living in the least deprived quintile had heard of the guidelines. Participants living in deprivation quintiles 3 and 4 were 1.70 and 1.95 times more likely, respectively, than those living in deprivation quintile 5 (least deprived) to have heard of the UK guidelines.

### 3.2. Awareness of the Physical Activity Recommendations

The MPA recommendation was the most definitively known recommendation of the three, with 30.7% of participants reporting knowing this, followed by only 13.3% and 13.4% knowing the VPA and muscle-strengthening recommendations, respectively (Supplementary Table S4). A vague awareness was reported by 42.4% for the MPA recommendation, by 24.4% for the VPA recommendation and by 19.1% for the muscle-strengthening recommendation. Thus, the muscle-strengthening recommendation was the least known, with more than two thirds (67.5%) not knowing it.

In bivariate analyses, an awareness of the MPA recommendation was significantly associated with sex, with more males reporting knowing it than females (Supplementary Table S4). Knowing the VPA recommendation was significantly associated with age, sex and disability status. The oldest and youngest age groups had a higher awareness, along with males and those living without a disability. Awareness of the muscle-strengthening recommendation was significantly associated with age, with awareness highest in the oldest age group. Knowing each recommendation was also significantly associated with having heard of the UK guidelines. As such, 54.0% of participants who had heard of the UK guidelines also knew the MPA recommendation, compared with only 24.2% of those who had not heard of the guidelines. Equivalent figures were 28.0% versus 9.2% for the VPA recommendation and 27.0% versus 9.6% for the muscle-strengthening recommendation.

The multivariate analyses explored independent associations between knowing the three recommendations (versus vague awareness or not knowing them) and participant socio-demographics and whether participants had heard of the UK guidance (Table 2). Having heard of the guidance remained strongly associated with knowing each of the three recommendations, with AORs (versus those that had not heard of the UK guidance) being 3.48 for the muscle-strengthening recommendation, 3.88 for the MPA recommendation and 4.03 for the VPA recommendation. No socio-demographic factors were independently associated with knowing the MPA recommendation. However, those aged 36–50 years were significantly less likely than 19–35-year-olds to know the VPA recommendation (AOR 0.54), females were significantly less likely to know the VPA recommendation than males (AOR 0.58) and participants with a disability were significantly less likely to know the muscle-strengthening recommendation than those without a disability (AOR 0.63).

### 3.3. Meeting the UK Physical Activity Recommendations

More than half of participants (55.1%) reported meeting the MVPA recommendation, and 29.5% of participants reported meeting the muscle-strengthening recommendation (Supplementary Table S5). The bivariate analyses found significant associations between meeting each recommendation and deprivation, disability status and knowing the recommendations. Thus, the proportion meeting each recommendation tended to increase as the deprivation level decreased; it was greater in those without a disability than those with a disability; and was greater in those who knew the recommendations. While meeting the muscle-strengthening recommendation was also significantly associated with having heard of the UK guidance, there was no significant association for the MVPA recommendation.

In the multivariate analyses, for meeting the MVPA recommendation, independent relationships were found with deprivation, disability status and awareness of the MVPA recommendation (Table 3). Participants living in the most deprived area (quintile 1) and those with a disability were significantly less likely to meet the MVPA recommendation compared with those living in the least deprived area (quintile 5) and those without a disability, respectively (AORs of 0.42 for both). Increasing knowledge of the individual MVPA recommendations was positively associated with meeting the MVPA recommendation, with those who knew one of the MVPA recommendations being 1.75 times more likely to meet the recommendation and those who knew both MVPA recommendations being 2.11 times more likely.

For meeting the muscle-strengthening recommendation, participants living in the more deprived quintiles (1 and 2) were significantly less likely than those living in the least deprived quintile to report meeting the recommendation (AORs of 0.59 and 0.55, respectively; Table 3). In addition, participants who reported knowing the muscle-strengthening recommendation were 2.07 times significantly more likely to report meeting the recommendation compared to those who did not know the recommendation.

## 4. Discussion

Given the significant investment required to develop and update physical activity guidelines, it is imperative to understand their reach to inform future updates and identify areas for focused dissemination to maximise their impact. Tailoring public health messages to target audiences has already been advocated for [19], but to achieve that we need to understand awareness gaps. The purpose of this study was to identify if differences existed in the awareness and adoption of the UK physical activity guidelines and its recommendations by socio-demographics and if awareness was associated with meeting the recommendations. To our knowledge, this is the first assessment of awareness in any of the four nations following the publication of the updated guidelines in 2019.

Only a fifth of participants (21.7%) reported having heard of the UK guidelines, with 30.7% reporting knowing the MPA recommendation, 13.3% knowing the VPA recommendation and 13.4% knowing the muscle-strengthening recommendation. This highlights the importance of the communication of the recommendations, not just the existence of the guidelines, and this should be measured accordingly in surveillance work. While the low knowledge of national guidelines found in Wales is consistent with that identified in studies in other countries such as the USA [13] and Canada [14], the awareness level was higher than that previously found in England, which is subject to the same UK physical activity guidelines as Wales [15]. However, the questions used to measure awareness were different between studies, and data were collected a decade apart. Given the focus that was placed on physical activity during the COVID-19 pandemic, this could have increased people’s awareness of physical activity in more recent years. Importantly, the proportion of participants reporting meeting the MVPA guidelines (55%) in this study is consistent with that found in a national survey conducted in 2022/2023 in Wales with over 6500 participants [26]. Whilst this adds strength, affirming the self-report surveillance with device-based measures (e.g., accelerometers) would provide a more accurate picture. While similar national surveillance data are not available to compare findings on adherence to the muscle-strengthening recommendation, the proportion reporting meeting this recommendation in our study (29.5%) was higher than that found in a systematic review of international studies (22.8%) [27]. Given that the guidelines place a balanced emphasis on the need to perform MVPA and muscle-strengthening activities, a step forward would be to have national surveillance of both. Additionally, vulnerable populations have previously been found to be less likely to report meeting the muscle-strengthening recommendation [27], and this pattern is found in this study with people living in more deprived areas less likely to report meeting the recommendation than those living in the most affluent area.

The existing literature reports contrasting patterns in relationships between socio-demographics and awareness of physical activity guidelines and recommendations. Only a small number of socio-demographic differences were found in the present study, thus advocating a need to continue to explore patterns in different geographic locations. For having heard of the UK guidelines, a significant difference was only found for deprivation, with awareness in the middle deprivation quintiles (3 and 4) being greater than in the least deprived quintile. The lack of differences in awareness between the most and least deprived quintiles could be explained by the increased touch points that people living in more deprived areas can have with healthcare professionals, which may be a route through which they are exposed to knowledge [28]. Our findings contrast with Dale and colleagues [14], who found no demographic differences in the awareness of the Canadian guidelines, and Chen and colleagues [13], who found older adults to be more aware than younger groups. However, given the time difference between when the data was collected between the studies, this highlights a need to continually review which population groups require the greatest level of intervention to increase awareness and engagement in the recommendations to achieve the desired behavioural outcomes, as it should not be assumed that the vulnerable groups remain consistent over time. Understanding the gaps enables practitioners to apply a proportionate universalist approach to delivery [29]. In addition, awareness in Canada may have changed by now, as in 2018 a common vision policy was introduced [30], the country’s first solo focused physical activity policy aimed at guiding people to be more physically active.

For specific recommendations, we found no socio-demographic differences in the knowledge of the MPA recommendation. However, males were significantly more likely than females to know the VPA recommendation, and those without a disability were significantly more likely to know the muscle-strengthening recommendation than those with a disability. While not all participants who reported having heard of the UK guidelines reported knowing each of the recommendations, a positive relationship was found between having heard of the guidance and knowing the recommendations and subsequently meeting the recommendations. This finding is supported by previous evidence, suggesting a relationship between knowledge and subsequent behaviour [16]. Public health guidance and recommendations for better health are not limited to physical activity. Studies on other guidelines such as eating five portions of fruit and vegetables a day have found an increased knowledge of the guidelines to be associated with an increased consumption of fruit and vegetables [31], showcasing knowledge as a predictor of adoption and adherence. This highlights the need to raise awareness of public health guidelines universally, as well as the obtainment of public acceptance of guidelines as a powerful driver for successful implementation [32,33].

Awareness of physical activity guidelines amongst the general public can be an evaluation metric of how effectively guidelines have been communicated [13], and questions exist on whether the public are more familiar with who published the guidelines as opposed to the actual recommendations. However, the proportion reporting knowing the MPA recommendation in this study was higher than the proportion that reported having heard of the guidelines. Whilst awareness alone will not be sufficient to increase physical activity levels, in health theories awareness is considered a building block of moving people from intention to performing a behaviour. Applying the Health Belief Model (HBM) [34] helps explain why awareness does not automatically translate into meeting the physical activity recommendations. Firstly, people may not perceive themselves as sufficiently at risk of inactivity (low perceived susceptibility) or may underestimate the health consequences (low perceived severity). Secondly, even when aware of the benefits, the knowledge may not sufficiently outweigh the perceived barriers to participating in the activity. Thirdly, differences in activity levels by socio-demographics could be explained by differences in people’s self-efficacy to address barriers and be active. Finally, awareness campaigns often act as cues to action, triggering intention formation but without the substance of addressing key determinants of behaviours. Hence, the impact of raising awareness alone has a limited scope for supporting positive behaviour change.

### 4.1. Implications for Practice

In Wales, the physical activity guidelines are one strategy within around 15 national policies and strategies to increase physical activity across the population [35]. The low awareness identified in this study highlights the need to include an accompanying communication and dissemination strategy for the public with the next update of the UK physical activity guidelines. The approach advocated for by Milton and colleagues [36], where an international set of guidelines is created, it would enable an international tracking of awareness that is comparable and generalisable across countries. One approach to dissemination could be through social media. In Wales, it is estimated that around 80% of adults (16+) use digital social media platforms, with no significant differences in access and usage between deprivation quintiles [37]. This provides a larger opportunity to implement digital health promotion campaigns. A core element that determines the precision and effective (audience) engagement levels of the digital campaign activity is audience segmentation [38]. The findings of our study could help inform the segmentation process for future awareness-raising campaigns, including the development of tailored messaging for specific groups, such as those with a low awareness for particular areas of the physical activity guidelines.

Previous UK guideline development committees and working groups have recognised the importance of communication strategies for the public. While there was an intention to produce guidelines for a public-facing communication strategy to support the implementation of the current UK guidelines, resource issues did not enable their production [20]. The integration of communication campaigns and evaluations of the implementation of guidelines is recommended [39]. To date, the centralised development and implementation of such a strategy and plan in the UK does not exist.

### 4.2. Limitations

This study is not without its limitations. A sampling framework was designed to achieve a nationally representative sample of the population of Wales that was aged 16+ years; however, optimal demographic groups for certain groups were not achieved, such as males and ethnic minority groups. Additionally, as the survey was conducted as part of a public health panel with no financial incentive for participation, both the participation and responses may be subject to sample selection bias. However, the similarity in physical activity levels to those found in other national surveys adds strength to this study. The questionnaire used prompted questions, which could lead to overestimations in the knowledge of the guidelines and recommendations. Earlier research has found prompted questions elicit higher self-reported knowledge than unprompted questions [40]. Finally, participants were asked to self-report whether they met the physical activity guidelines. However, a more robust approach would involve using a device-based measure (e.g., accelerometers and heart rates) to assess physical activity levels, as accurately distinguishing between intensity levels without a device is difficult.

## 5. Conclusions

The identification of relationships between the awareness and adoption of the guidelines by different socio-demographic variables can aid the planning of campaigns and implementation of current and future guidelines. The findings highlight the importance of resourcing a public communication strategy to coincide with the delivery of guidelines to support their dissemination, as ultimately the public are the end-users of the knowledge provided through the guidelines. This finding can be applied to the implementation of other public health guidelines—not just those relating to physical activity.

## Figures and Tables

**Table 1:** Bivariate relationships and adjusted odds ratios (AORs) for reporting having heard of CMO’s physical activity guidelines by participant demographics.

Categories		Yes (%)	No (%)	Yes, Heard of the CMO Guidelines	
				AOR (95% CI)	*p*
All		21.7	78.3		
Age group (years)	19–35	22.4	77.6	Ref	0.564
	36–50	22.4	77.6	0.95 (0.61–1.48)	0.837
	51–64	18.5	81.5	0.75 (0.47–1.19)	0.222
	65+	23.4	76.6	0.99 (0.64–1.54)	0.970
	*X*2		2.015		
	*p*		0.569		
Sex	Male	21.8	78.2	Ref	
	Female	21.6	78.4	0.95 (0.67–1.34)	0.774
	*X*2		0.005		
	*p*		0.946		
Deprivation quintile	1—Most	14.8	85.2	0.84 (0.47–1.48)	0.546
	2	21.3	78.7	1.34 (0.80–2.24)	0.265
	3	25.6	74.4	1.70 (1.05–2.75)	0.030
	4	28.4	71.6	1.95 (1.22–3.10)	0.005
	5—Least	16.8	83.2	Ref	0.005
	*X*2		14.990		
	*p*		0.005		
Disability status	No	21.6	78.4	Ref	
	Yes	21.8	78.2	1.07 (0.78–1.48)	0.656
	*X*2		0.006		
	*p*		0.939		

CMO, Chief Medical Officer; 95% CI, 95% confidence interval; AOR, adjusted odds ratio; and Ref, reference category.

**Table 2:** Adjusted odds ratios (AORs) for reporting knowing each of the three physical activity recommendations by participant demographics and awareness of the guidelines.

Categories		MPA		VPA		Muscle-Strengthening	
		AOR (95% CI)	*p*	AOR (95% CI)	*p*	AOR (95% CI)	*p*
Age group (years)	19–35	Ref	0.116		0.068		0.059
	36–50	0.67 (0.44–1.02)	0.062	0.54 (0.30–0.97)	0.038	0.68 (0.38–1.21)	0.188
	51–64	1.03 (0.68–1.57)	0.886	0.65 (0.36–1.17)	0.153	0.81 (0.45–1.45)	0.477
	65+	1.03 (0.69–1.54)	0.889	1.02 (0.61–1.70)	0.951	1.36 (0.81–2.28)	0.248
Sex	Female	0.74 (0.55–1.02)	0.062	0.58 (0.39–0.87)	0.009	0.95 (0.63–1.44)	0.813
Deprivation quintile	1—Most	0.92 (0.58–1.47)	0.739	0.97 (0.51–1.81)	0.913	0.94 (0.48–1.85)	0.867
	2	0.73 (0.46–1.16)	0.181	0.74 (0.39–1.41)	0.370	1.32 (0.72–2.43)	0.373
	3	0.69 (0.45–1.08)	0.106	0.74 (0.41–1.35)	0.323	0.99 (0.55–1.80)	0.977
	4	0.93 (0.61–1.41)	0.721	0.97 (0.55–1.69)	0.904	1.16 (0.66–2.05)	0.610
	5—Least	Ref	0.423		0.778		0.826
Disability status	Yes	0.81 (0.60–1.08)	0.154	0.67 (0.44–1.01)	0.056	0.63 (0.42–0.95)	0.026
Heard of CMO guidelines	Yes	3.88 (2.80–5.37)	<0.001	4.03 (2.69–6.03)	<0.001	3.48 (2.34–5.17)	<0.001

95% CI, 95% confidence interval; AOR, adjusted odds ratio; Ref, reference category; MPA, moderate physical activity; VPA, vigorous physical activity; and CMO, Chief Medical Officer. For binary variables, reference categories were male (for sex) and no (for disability status and heard of CMO guidance).

**Table 3:** Adjusted odds ratios (AORs) of reporting meeting the CMO’S physical activity recommendations by participants’ socio-demographics and awareness of guidelines and recommendations.

Categories		Meet the MVPA Recommendation		Meet the Muscle-Strengthening Recommendation	
		AOR (95% CI)	*p*	AOR (95% CI)	*p*
Age group (years)	19–35	Ref	0.703		0.502
	36–50	0.95 (0.64–1.39)	0.779	1.00 (0.66–1.51)	0.987
	51–64	1.17 (0.79–1.73)	0.447	1.31 (0.86–1.99)	0.210
	65+	0.97 (0.66–1.43)	0.969	1.04 (0.69–1.57)	0.849
Sex	Female	0.99 (0.74–1.33)	0.942	0.97 (0.71–1.32)	0.831
Deprivation quintile	1—Most	0.42 (0.27–0.66)	<0.001	0.59 (0.36–0.94)	0.028
	2	0.66 (0.43–1.01)	0.055	0.55 (0.34–0.87)	0.011
	3	0.86 (0.57–1.30)	0.466	0.94 (0.62–1.42)	0.769
	4	0.70 (0.46–1.04)	0.080	0.83 (0.55–1.25)	0.377
	5—Least	Ref	0.002		0.037
Disability status	Yes	0.42 (0.32–0.55)	<0.001	0.82 (0.61–1.09)	0.171
Heard of CMO guidelines	Yes	1.05 (0.75–1.48)	0.772	1.26 (0.89–1.80)	0.189
Knew MVPA recommendation	No	Ref	0.002		0.457
	Yes, one	1.75 (1.22–2.51)	0.002	0.98 (0.67–1.43)	0.982
	Yes, both	2.11 (1.22–3.64)	0.008	1.35 (0.80–2.27)	0.262
Knew muscle-strengthening recommendation	Yes	1.07 (0.65–1.75)	0.787	2.07 (1.29–3.32)	0.002

95% CI, 95% confidence interval; AOR, adjusted odds ratio; Ref, reference category; MVPA, moderate-to-vigorous physical activity; and CMO, Chief Medical Officer. For binary variables, reference categories were male (for sex) and no (for disability status, heard of CMO guidance and knew muscle-strengthening recommendation).

## Data Availability

The datasets used and analysed during the current study are available from the corresponding author on reasonable request.

## Supplementary Materials

The following supporting information can be downloaded at https://www.mdpi.com/article/10.3390/ijerph23010005/s1: Supplementary Table S1. Variables included in the analysis; Supplementary Table S2. Demographics of the survey sample; Supplementary Table S3. Bivariate relationship of outcome variables by ethnicity; Supplementary Table S4. Bivariate relationship of knowledge of the three physical activity recommendations; and Supplementary Table S5. Bivariate relationship of participants reporting meeting the physical activity recommendations.

## References

[1] World Health Organization The Top 10 Causes of Death. https://www.who.int/news-room/fact-sheets/detail/the-top-10-causes-of-death.

[2] Lee I., Shiroma E., Lobelo F., Puska P., Blair S., Katzmarzyk P.T., Lancet Physical Activity Series Working Group (2012). Effect of physical inactivity on major non-communicable diseases worldwide: An analysis of burden of disease and life expectancy. Lancet.

[3] Katzmarzyk P. (2023). Expanding our understanding of the global impact of physical inactivity. Lancet.

[4] Strain T., Flaxman S., Guthold R., Semenova E., Cowan M., Riley L.M., Bull F.C., A Stevens G., Raheem R.A., Agoudavi K. (2024). National, regional, and global trends in insufficient physical activity among adults from 2000 to 2022: A pooled analysis of 507 population-based surveys with 5.7 million participants. Lancet Glob. Health.

[5] World Health Organization Global Action Plan on Physical Activity 2018–2030. More Active People for a Healthier World. https://iris.who.int/bitstream/handle/10665/272722/9789241514187-eng.pdf?sequence=1.

[6] Department of Health and Social Care, Welsh Government, Department of Health Northern Ireland, The Scottish Government UK Chief Medical Officers Physical Activity Guidelines. https://assets.publishing.service.gov.uk/media/5d839543ed915d52428dc134/uk-chief-medical-officers-physical-activity-guidelines.pdf.

[7] Blair S.N., LaMonte M.J., Nichaman M.Z. (2004). The evolution of physical activity recommendations: How much is enough?. Am. J. Clin. Nutr..

[8] World Health Organization Global Recommendations on Physical Activity for Health. https://www.paho.org/sites/default/files/WHO-Global-recommendations-physical-activity-2010.pdf.

[9] World Health Organization (2020). WHO Guidelines on Physical Activity and Sedentary Behaviour. https://iris.who.int/bitstream/handle/10665/336656/9789240015128-eng.pdf?sequence=1&isAllowed=y.

[10] Bull F.C., Al-Ansari S.S., Biddle S., Borodulin K., Buman M.P., Cardon G., Carty C., Chaput J.-P., Chastin S., Chou R. (2020). World Health Organization 2020 guidelines on physical activity and sedentary behaviour. Br. J. Sports Med..

[11] Department of Health and Social Care UK Physical Activity Guidelines. https://www.gov.uk/government/publications/uk-physical-activity-guidelines.

[12] Gelius P., Tcymbal A., Abu-Omar K., Mendes R., Morais S.T., Whiting S., Breda J. (2022). Status and contents of physical activity recommendations in European Union countries: A systematic comparative analysis. BMJ Open.

[13] Chen T.J., Whitfield G.P., Watson K.B., Fulton J.E., Ussery E.N., Hyde E., Rose K. (2023). Awareness and knowledge of the physical activity guidelines for americans, 2nd edition. J. Phys. Act. Health.

[14] Dale L.P., LeBlanc A.G., Orr K., Berry T., Deshpande S., Latimer-Cheung A.E., O’rEilly N., Rhodes R.E., Tremblay M.S., Faulkner G. (2016). Canadian physical activity guidelines for adults: Are Canadians aware?. Appl. Physiol. Nutr. Metab..

[15] Knox E.C.L., Esliger D.W., Biddle S.J.H., Sherar L.B. (2013). Lack of knowledge of physical activity guidelines: Can physical activity promotion campaigns do better?. BMJ Open.

[16] Wafi A.M., Wadani S.N., Daghriri Y.Y., Alamri A.I., Zangoti A.M., Khiswi A.A., Al-Ebrahim E.Y., Jesudoss H.J., Alharbi A.A. (2024). Awareness and knowledge of the physical activity guidelines and their association with physical activity levels. Sports.

[17] Tajima T., Harada K., Oguma Y., Sawada S.S. (2023). Does health literacy moderate the psychological pathways of physical activity from guideline awareness to behaviour? A multi-group structural equation modelling. BMC Public Health.

[18] Nobles J., Thomas C., Gross Z.B., Hamilton M., Trinder-Widdess Z., Speed C., Gibson A., Davies R., Farr M., Jago R. (2020). “Let’s talk about physical activity”: Understanding the preferences of under-served communities when messaging physical activity guidelines to the public. Int. J. Environ. Res. Public Health.

[19] Williamson C., Baker G., Mutrie N., Niven A., Kelly P. (2020). Get the message? A scoping review of physical activity messaging. Int. J. Behav. Nutr. Phys. Act..

[20] Milton K., Bauman A.E., Faulkner G., Hastings G., Bellew W., Williamson C., Kelly P. (2020). Maximising the impact of global and national physical activity guidelines: The critical role of communication strategies. Br. J. Sports Med..

[21] Jones S.M., Porroche-Escudero A., Shearn K., Hunter R.F., Garcia L. (2024). Thinking about inequalities in physical activity as an emergent feature of complex systems. Int. J. Behav. Nutr. Phys. Act..

[22] Sharp C., Hughes K., Hill R. (2022). Time to Talk Public Health: Creation and Establishment of a Nationally Representative Panel. Protocol. https://phw.nhs.wales/topics/time-to-talk-public-health/time-to-talk-public-health-panel-publications/time-to-talk-public-health-protocol/.

[23] StatsWales (2019). Welsh Index of Multiple Deprivation (WIMD) 2019. https://statswales.gov.wales/Catalogue/Community-Safety-and-Social-Inclusion/Welsh-Index-of-Multiple-Deprivation.

[24] Office for National Statistics Population Estimates for the UK, England and Wales, Scotland and Northern Ireland: Mid-2020. https://www.ons.gov.uk/peoplepopulationandcommunity/populationandmigration/populationestimates/bulletins/annualmidyearpopulationestimates/mid2020.

[25] Office for National Statistics Measuring Disability: Comparing Approaches. https://www.ons.gov.uk/peoplepopulationandcommunity/healthandsocialcare/disability/articles/measuringdisabilitycomparingapproaches/2019-08-06.

[26] Welsh Government National Survey for Wales Headline Results: April 2022 to March 2023. https://www.gov.wales/national-survey-wales-headline-results-april-2022-march-2023-html.

[27] Ren Z., Zhang Y., Drenowatz C., Eather N., Hong J., Wang L., Yan J., Chen S. (2024). How many adults have sufficient muscle-strengthening exercise and the associated factors: A systematic review consisting of 2,629,508 participants. J. Exerc. Sci. Fit..

[28] Barlow P., Mohan G., Nolan A., Lyons S. (2021). Area-level deprivation and geographic factors influencing utilisation of general practitioner services. SSM Popul. Health.

[29] Marmot M., Allen J., Boyce T., Goldblatt P., Morrison J. Fair Society, Healthy Lives. The Marmot Review. Institute for Health Equity. https://www.instituteofhealthequity.org/resources-reports/fair-society-healthy-lives-the-marmot-review/fair-society-healthy-lives-full-report-pdf.pdf.

[30] Public Health Agency of Canada A Common Vision for Increasing Physical Activity and Reducing Sedentary Living in Canada. Let’s Get Moving. https://www.canada.ca/en/public-health/services/publications/healthy-living/lets-get-moving.html.

[31] Appleton K.M., Krumplevska K., Smith E., Rooney C., McKinley M.C., Woodside J.V. (2017). Low fruit and vegetable consumption is associated with low knowledge of the details of the 5-a-day fruit and vegetables message in the UK: Findings from two cross-sectional questionnaire studies. J. Hum. Nutr. Diet..

[32] Milton K., Buse K., Bull F. (2025). Powerful actors who undermine physical activity policy must be challenged. BMJ.

[33] Reynolds J., Likki T., Habersaat K.B. (2024). Measuring and Maximizing Public Support for Health Policies: Behavioural and Cultural Insights Policy Brief Series.

[34] Chatzisarantia N.L.D., Kamarova V., Kawabata M., Hagger M.S., Eklund R.C., Tenenbaun G. (2014). Health Belief Model. Encyclopedia of Sport and Exercise Psychology.

[35] Sharp C.A., Mackintosh K.A., Willmot R., Hughes R., McNarry M., Milton K. (2022). National Policy Response to the United Nations Sustainable Development Goals: A Physical Activity Case Study of Wales. J. Phys. Act. Health.

[36] Milton K., Hanson C.L., Pearsons A., Chou R., Stamatakis E. (2024). A narrative review of global and national physical activity and sedentary behaviour guidelines development processes—The guidelines standards (GUS) project. Prev. Med..

[37] Song J., Sharp C.A., Davies A. Population health in a digital age. In *Patterns in the Use of Social Media in Wales*; Public Health Wales: Cardiff, UK; Bangor University: Bangor, UK, 2020. https://phw.nhs.wales/topics/digital-technology-and-health/population-health-in-a-digital-age1/.

[38] Evans W.D., Thomas C.N., Favatas D., Smyser J., Briggs J. (2020). Digital segmentation of priority populations in public health. Health Educ. Behav..

[39] Kauffeldt K.D., Latimer-Cheung A.E., Faulkner G., Brouwers M.C., Jones R., Lane K.N., Weston Z.J., Morgan T.L., Varkul O., Tomasone J.R. (2025). The longitudinal evaluation of the Canadian 24-houor movement guidelines for adults: Lessons learned and considerations for future research. Appl. Physiol. Nutr. Metab..

[40] Cameron C., Craig C.L., Bull F.C., Bauman A. (2007). Canada’s physical activity guides: Has their release had an impact. Appl. Phys. Nutr. Metab..

