# Supplementary material for: The Awareness and Adoption of UK Physical Activity Guidelines by Socio-Demographics: A National Cross-Sectional Survey in Wales
Source: Int J Environ Res Public Health. 2025 Dec 19;23(1):5. doi: 10.3390/ijerph23010005 (PMC12841382; doi:10.3390/ijerph23010005)
Supplement: Supplementary file 1 [file ijerph-23-00005-s001.zip › ijerph-4008586-supplementary.pdf]

Supplementary Table S1. Variables included in the analysis

	Question *(response options)*	Qualifying response
Knowledge of UK CMO's physical activity guidelines	Before today's survey, had you heard of the UK Chief Medical Officers' physical activity guidelines? (Yes; No; Prefer not to say)	Yes
Knowledge of individual recommendations in the UK CMO's physical activity guidelines	Which of the following guideline recommendations for adults have you heard of? (Yes – I knew this recommendation; Yes – I had a vague idea of this recommendation; No – I did not know this recommendation; Don't know; Prefer not to say)	Yes – I knew this recommendation
	Adults should do at least 150 minutes of moderate physical activity each week, such as brisk walking or light effort cycling	
	Adults should do at least 75 minutes of vigorous physical activity each week, such as running or fast cycling	
	Adults should do muscle-strengthening activities on at least 2 days per week	
Perception of meeting the UK CMO's physical activity recommendations	Over the past week, have you done either of the following? (Yes; No; Don't know; Prefer not to say)	Yes
	At least 150 minutes of moderate physical activity or 75 minutes of vigorous physical activity per week	
	Muscle-strengthening activities on at least 2 days per week	

Supplementary Table S2. Demographics of the survey sample

		n	%
**All**		972	100%
**Age group (years)**	19-35	219	22.5%
	36-50	255	26.2%
	51-64	233	24.0%
	65+	265	27.3%
**Sex**	Male	293	30.1%
	Female	679	69.9%
**Deprivation quintile**	1 - Most	162	16.7%
	2	178	18.3%
	3	203	20.9%
	4	215	22.1%
	5 - Least	214	22.0%
**Ethnicity**	White (including ethnic minority white)	949	97.6%
	Other than white	23	2.4%
**Disability status**	No	532	54.7%
	Yes	440	45.3%

Supplementary Table S3. Bivariate relationship of outcome variables by ethnicity

		White (including minority white; %)	Other than white (%)
Heard of the CMO guidelines	Yes	21.7	21.7
	No	78.3	78.3

	X2		0.000
	p		0.997
Knew MPA recommendation	Yes - knew it	30.5	39.1
	Yes - vaguely	42.5	39.1
	No / Don't know	27.1	21.7
	X2		0.848
	p		0.654
Knew VPA recommendation	Yes - knew it	13.1	21.7
	Yes - vaguely	24.6	17.4
	No / Don't know	62.4	60.9
	X2		1.753
	p		0.416
Knew muscle-strengthening recommendation	Yes - knew it	13.4	13.0
	Yes - vaguely	18.9	30.4
	No / Don't know	67.8	56.5
	X2		1.993
	p		0.369
Meet the MVPA recommendation	Yes	55.0	60.9
	No	45.0	39.1
	X2		0.312
	p		0.576
Meet the muscle-strengthening recommendation	Yes	29.8	17.4
	No	70.2	82.6
	X2		1.667
	p		0.197

CMO, Chief Medical Officer; MPA, Moderate physical activity; VPA, Vigorous physical activity; MVPA, Moderate-to-vigorous physical activity.

Supplementary Table S4. Bivariate relationship of knowledge of the three physical activity recommendations

		**MPA recommendation**			**VPA recommendation**			**Muscle-strengthening recommendation**		
		**Yes - knew it (%)**	**Yes - vaguely (%)**	**No / Don't know (%)**	**Yes - knew it (%)**	**Yes - vaguely (%)**	**No / Don't know (%)**	**Yes - knew it (%)**	**Yes - vaguely (%)**	**No / Don't know (%)**
**All**		30.7	42.4	27.0	13.3	24.4	62.3	13.4	19.1	67.5
**Age group (years)**	19-35	32.4	38.4	29.2	16.0	21.9	62.1	14.2	16.4	69.4
	36-50	24.7	47.5	27.8	9.4	23.9	66.7	10.2	15.3	74.5
	51-64	31.3	43.3	25.3	10.3	23.2	66.5	10.7	21.5	67.8
	65+	34.3	40.0	25.7	17.4	27.9	54.7	18.1	23.0	58.9
	X2			8.052			**14.922**			**18.142**
	p			0.234			**0.021**			**0.006**
**Sex**	Male	35.8	35.2	29.0	18.4	27.3	54.3	14.7	20.5	64.8
	Female	28.4	45.5	26.1	11.0	23.1	65.8	12.8	18.6	68.6
	X2			**9.502**			**14.269**			1.360
	p			**0.009**			**0.001**			0.507
**Deprivation**	1 - Most	30.2	33.3	36.4	13.0	15.4	71.6	10.5	14.2	75.3

**quintile**	2	27.0	44.4	28.7	10.7	27.2	62.4	14.6	20.2	65.2
	3	28.1	45.8	26.1	12.3	26.1	61.6	13.3	25.5	61.1
	4	34.4	41.9	23.7	15.8	23.7	60.5	15.8	15.8	68.4
	5 - Least	32.7	44.9	22.4	14.0	28.0	57.9	12.1	19.2	68.7
	X2			14.799			12.496			13.188
	p			0.063			0.130			0.106
**Disability status**	No	32.9	43.0	24.1	15.6	26.7	57.7	15.6	17.9	66.5
	Yes	28.0	41.6	30.5	10.5	21.6	68.0	10.7	20.7	68.6
	X2			5.690			**11.433**			5.519
	p			0.058			**0.003**			0.063
**Heard of CMO guidance**	No	24.2	43.0	32.9	9.2	22.3	68.5	9.6	16.0	74.4
	Yes	54.0	40.3	5.7	28.0	31.8	40.3	27.0	30.3	42.7
	X2			**93.514**			**70.868**			**79.773**
	p			**<0.001**			**<0.001**			**<0.001**

MPA, moderate physical activity; VPA, vigorous physical activity; CMO, Chief Medical Officer.

Supplementary Table S5. Bivariate relationship of participants reporting meeting the physical activity recommendations

**Categories**			**Meet the MVPA recommendation**		**Meet the muscle-strengthening recommendation**	
			**Yes**	**No**	**Yes**	**No**
**All**			55.1	44.9	29.5	70.5
**Age group (years)**	19-35		54.8	45.2	27.9	72.1
	36-50		52.9	47.1	27.5	72.5
	51-64		56.7	43.3	32.2	67.8
	65+		56.2	43.8	30.6	69.4
		X2		0.851		1.754
		p		0.837		0.625
**Sex**	Male		57.3	42.7	30.7	69.3
	Female		54.2	45.8	29.0	71.0
		X2		0.816		0.285
		p		0.366		0.593
**Deprivation quintile**	1 - Most		41.4	58.6	22.8	77.2
	2		50.0	50.0	22.5	77.5
	3		59.1	40.9	33.5	66.5
	4		56.7	43.3	31.6	68.4
	5 - Least		64.5	35.5	34.6	65.4
		X2		**23.417**		**12.359**
		p		**<0.001**		**0.015**
**Disability status**	No		65.2	34.8	32.3	67.7
	Yes		43.0	57.0	26.1	73.9
		X2		**48.290**		4.441
		p		**<0.001**		**0.035**
**Heard of CMO guidelines**	No		53.7	46.3	27.6	72.4
	Yes		60.2	39.8	36.5	63.5
		X2		2.774		**6.285**
		p		0.096		**0.012**

**Knew MVPA recommendation**	Did not know this	49.9	50.1	26.4	73.6
	Yes - knew one	63.3	36.7	30.9	69.1
	Yes - knew both	70.9	29.1	45.5	54.5
	X2		**23.815**		**16.657**
	p		**<0.001**		**<0.001**
**Knew muscle-strengthening recommendation**	Did not know this	53.2	46.8	26.7	73.3
	Yes - knew this	67.7	32.3	47.7	52.3
	X2		**9.553**		**23.799**
	p		**0.002**		**<0.001**

MVPA, Moderate-to-vigorous physical activity; CMO, Chief Medical Officer.
